# In Vitro and In Silico Investigation of BCI Anticancer Properties and Its Potential for Chemotherapy-Combined Treatments

**DOI:** 10.3390/cancers15184442

**Published:** 2023-09-06

**Authors:** Beata Marciniak, Mateusz Kciuk, Somdutt Mujwar, Rajamanikandan Sundaraj, Karol Bukowski, Renata Gruszka

**Affiliations:** 1 Department of Molecular Biotechnology and Genetics, University of Lodz, Banacha 12/16, 90-237 Lodz, Poland; mateusz.kciuk@edu.uni.lodz.pl (M.K.); karol.bukowski@edu.uni.lodz.pl (K.B.); renata.gruszka@biol.uni.lodz.pl (R.G.); 2 Doctoral School of Exact and Natural Sciences, University of Lodz, 90-237 Lodz, Poland; 3 Chitkara College of Pharmacy, Chitkara University, Rajpura 140401, Punjab, India; somduttmujwar@gmail.com; 4 Centre for Drug Discovery, Department of Biochemistry, Karpagam Academy of Higher Education, Coimbatore 641021, Tamil Nadu, India; rajamanikandan.sundararaj@kahedu.edu.in

**Keywords:** apoptosis, BCI, cytotoxicity, dual specificity phosphatase 6 (DUSP6), irinotecan, oxaliplatin

## Abstract

**Simple Summary:**

BCI is an allosteric inhibitor of DUSP6 phosphatase, which is a negative regulator of MAPK kinases involved in numerous cellular processes such as proliferation, differentiation, and survival. These studies were designed to test the anticancer potential of BCI in colorectal cancer (CRC) cells and assess its interaction with chemotherapuetics such as irinotecan and oxaliplatin. In silico investigations were performed to indicate new possible molecular targets, which in the future will help toward understanding the precise mechanism of action.

**Abstract:**

Background: DUSP6 phosphatase serves as a negative regulator of MAPK kinases involved in numerous cellular processes. BCI has been identified as a potential allosteric inhibitor with anticancer activity. Our study was designed to test the anticancer properties of BCI in colon cancer cells, to characterize the effect of this compound on chemotherapeutics such as irinotecan and oxaliplatin activity, and to identify potential molecular targets for this inhibitor. Methods: BCI cytotoxicity, proapoptotic activity, and cell cycle distribution were investigated in vitro on three colon cancer cell lines (DLD1, HT-29, and Caco-2). In silico investigation was prepared to assess BCI drug-likeness and identify potential molecular targets. Results: The exposure of colorectal cancer cells with BCI resulted in antitumor effects associated with cell cycle arrest and induction of apoptosis. BCI exhibited strong cytotoxicity on DLD1, HT-29, and Caco-2 cells. BCI showed no significant interaction with irinotecan, but strongly attenuated the anticancer activity of oxaliplatin when administered together. Analysis of synergy potential further confirmed the antagonistic interaction between these two compounds. In silico investigation indicated CDK5 as a potential new target of BCI. Conclusions: Our studies point to the anticancer potential of BCI but note the need for a precise mechanism of action.

## 1. Introduction

According to the World Health Organization, cancer is the leading first or second cause of premature mortality among people in highly developed countries. Inflammatory diseases, sedentary work style, lack of physical activity, unhealthy diet, excessive alcohol consumption, and tobacco smoking are the main risk factors for colorectal cancer (CRC). Increasing public awareness and widespread screening approaches are helping to reduce the mortality rate of the disease. However, still it remains listed as one of the most common (third) and deadly (second) cancers in the world. In 2020, there were more than 1.9 million incidences of CRC, mostly in Europe [1,2]. In 2023, it is predicted that there will be approximately 153,020 newly diagnosed cases of CRC in the United States, with an estimated 52,550 deaths due to this disease, of which approximately 7% (3750) will be individuals younger than 50 years of age [3].

CRC treatment is a complex subject due to its intratumoral heterogeneity, individual variability, and extreme genomic instability, while surgical treatment and chemotherapy remain the primary treatment option. The development of personalized therapies with fewer side effects requires understanding the source of these variables at both the primary and metastatic tumor levels. Dual specificity phosphatase 6 (DUSP6) is a member of the phosphatase subfamily (MAPK phosphatases, MKPs), which negatively regulates the mitogen-activated protein kinases (MAPKs). An extracellular signal-regulated kinase (ERK) involved in cell proliferation, differentiation, and survival ability constitutes the primary target of the DUSP6 protein. ERK plays an important role in both normal and tumor-transformed cells by transmitting proliferation stimuli in response to growth factors, hormones, or mitogens. Alterations of RAS/RAF/MEK/ERK signaling pathways have been described in many types of cancer [4,5,6,7]. Upregulation of upstream or downstream components in the pathway leads to its constitutive activation, which confers to tumor formation, invasion, and metastasis. Epidermal growth factor receptor (EGFR) overexpression and *Ras* or *B-Raf* mutations are examples of factors that lead to the overactivation of the pathway [8]. However, ERK-mediated signaling can lead to senescence in cells with *Ras* oncogene expression through the degradation of proteins involved in the cell cycle regulation. The attenuation of the ERK signaling on different levels may result in the transformation of mouse and human primary fibroblasts [5]. Under certain circumstances, ERK activity can also lead to cell death through sensitization to chemotherapeutic drugs or ionizing radiation (IR) [9].

Pathway dysregulation involving phosphatases activity is increasingly investigated in the context of cancer. MAPK dephosphorylation causes signal transduction to stop, therefore lack of MKPs activities can be linked to cancer development. The vast majority of evidence points to an association between MKPs deficiency and tumor advancement, poor differentiation, and invasiveness [8]. On the other hand, some studies have shown increased levels of these proteins in correlation with tumor development, drug resistance, and a poor prognosis for the patients [8,10]. In several types of cancers such as pre-B acute lymphoblastic leukemia (ALL) or malignant peripheral nerve sheath tumor (MPNST) where DUSP1/6 upregulation is observed, genetic or drug inhibition suppresses tumor growth [11,12]. In such instances, it is hypothesized that DUSPs facilitate the adaptive response of tumors to excessive levels of growth-factor signaling. Deficiency of these enzymes can lead to the activation of effectors such as cellular tumor antigen p53 (TP53), ATM serine/threonine kinase (ATM), and checkpoint kinases (CHK1/2), which are capable of impeding cell growth and/or triggering apoptosis [11].

**Figure 1:**
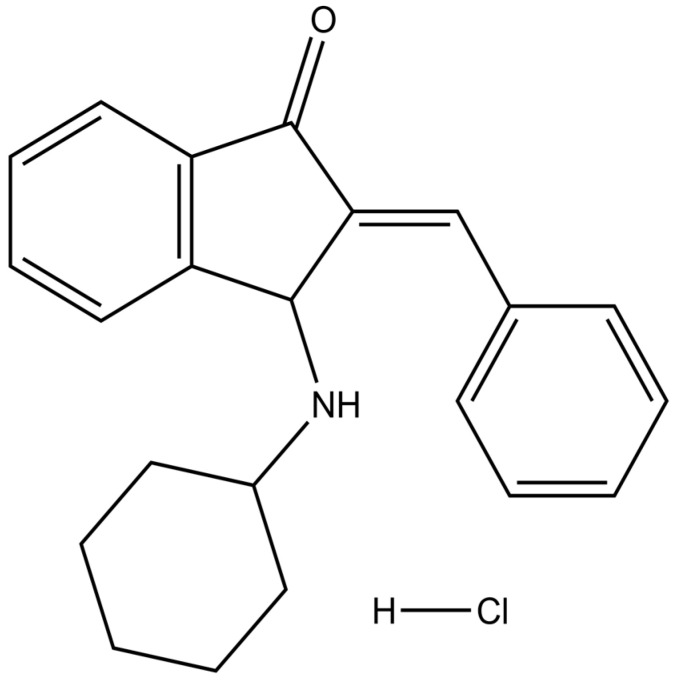
Chemical structure of BCI-hydrochloride.

BCI ((E)-2-benzylidene-3-(cyclohexylamino)-2,3-dihydro-1H-inden-1-one) is a small molecule that demonstrates the capacity for highly specific inhibition of the phosphatase activity of DUSP6 (Figure 1). Specifically, it binds to a less active form of DUSP6 and does so in an allosteric manner [13]. The primary mechanism through which BCI exhibits its anticancer effect is predominantly attributed to the inhibition of DUSP6 phosphatase. The application of BCI has been demonstrated to effectively suppress the proliferation, migration, and invasion of gastric cancer cells as well as sensitize them to cisplatin [10]. Some studies have provided evidence that BCI has yielded favorable outcomes when employed on various types of tumor cells through the DUSP6-dependent pathway, while others suggested that the anticancer activity may not result from DUSP6 inhibition [14]. However, the precise mechanism of this remains unknown.

In this in vitro study, we evaluated the impact of BCI on colon cancer cells in terms of its cytotoxicity and its ability to induce apoptotic cell death. Additionally, we examined the impact of BCI on the anticancer properties of chemotherapeutic agents such as irinotecan and oxaliplatin, and an ATM kinase inhibitor (KU60019) that is currently being evaluated in clinical trials. In parallel, we performed computational studies to identify potential mechanisms of BCI action that are not limited to DUSP6 inhibition and warrant further investigation.

## 2. Material and Methods

### 2.1. Chemicals

BCI hydrochloride, irinotecan hydrochloride, oxaliplatin, KU60019, and camptothecin were purchased from MedChemExpress LLC (TriMen Chemicals, Lodz, Poland). Except for oxaliplatin, each of these compounds was dissolved in DMSO; in the case of oxaliplatin, the solvent was H_2_O. Culture media, phosphate-buffered saline (PBS), penicillin–streptomycin mixture, and trypsin/EDTA were provided by Biowest (CytoGen, Zgierz, Poland). Fetal bovine serum (FBS), DMSO, MTT, and propidium iodide were purchased from Merck/Sigma Aldrich Chemical Co (Burlington, MA, USA). Caspase-Glo^®^ 3/7 Assay System was provided by Promega (Madison, WI, USA). Alexa Fluor^®^ 488 Annexin V/Dead Cell Apoptosis Kit was purchased from Invitrogen (Eugene, OR, USA).

### 2.2. Cell Culture

The study used three colon cancer adherent cell lines. DLD1 were purchased from Horizon Discovery group (Cambridge, UK) and cultured on RPMI 1640, supplemented with 10% (*v/v*) FBS and 1% (*v/v*) antibiotics (penicillin–streptomycin) mix solution. HT-29 and Caco-2 cell lines were supplied by American Type Culture Collection (Rockville, MD, USA) and cultivated, respectively, on RPMI 1640 and DMEM medium with standard supplementation (10% FBS and 1% of antibiotic mix). Cells were cultured in a 5% CO_2_ incubator at 37 °C and passaged (twice a week) with 0.25% trypsin/EDTA to maintain stable cell growth. All cells were routinely tested for Mycoplasma contamination using the MycoBlue Mycoplasma Detector (Vazyme Biotech, Nanjing, China) before each experiment.

In each experiment, cells were seeded at an optimal density specific to the cell line and duration of the experiment, as specified in Table 1.

### 2.3. Cytotoxicity Study

The MTT test was used to evaluate the cytotoxicity of the examined compounds and their combinations. This microplate assay is based on cellular redox potential. In living, metabolically active cells, mitochondrial succinate dehydrogenase enzymes lead to the reduction of the water-soluble tetrazolium salt (3-(4,5-dimethylthiazol-2-yl)-2,3-diphenyltetrazolium bromide—MTT) to water-insoluble purple formazan crystals. When dissolved in dimethylsulfoxide (DMSO), colorful solutions are formed so that their absorbance can be measured spectrophotometrically at λ = 570 nm. The method allowed for quantitative measurement of cell viability and proliferation.

Briefly, cells were seeded in standard 96-well plates at density depending on the cell line (Table 1 for adaptive incubation (37 °C, 5% CO_2_)). After 24 h, cells were treated with the investigated compounds over a wide range of concentrations (from 0.1 to 500 µM) and incubated (37 °C, 5% CO_2_) for 24 or 72 h. Following incubation, 20 µL/well of fresh MTT reagent was added at a concentration of 5 mg/mL. After 3 h, the medium was replaced with an organic solvent, and the absorbance of the solution was measured using a microplate reader, Power Wave XS BioTek Instruments, Inc. (Winooski, VT, USA).

### 2.4. Caspase 3/7 Activity Assay

The activity of caspase 3/7 enzymes was determined by using Caspase-Glo^®^ 3/7 Assay System following the manufacturer’s protocol. The principle of the method is to deliver to the cell’s environment a proluminescent caspase-3/7 DEVD-aminoluciferin substrate. Following cell lysis, the released active apoptotic enzymes proteolytically cleave the substrate, thus releasing free aminoluciferin. The thermostable luciferase supplied with the reagent then uses aminoluciferin to produce a luminescent signal. Thus, signal intensity is proportional to the caspase 3/7 activity.

Cells were seeded in 96-well white bottom plates (Table 1) for 24 h accommodation time (standard conditions: 37 °C, 5% CO_2_). Afterward, BCI was added for another 6 or 24 h at three concentrations selected based on the values obtained in the MTT test: 1, 2, and 4 μM. The untreated cell constituted a negative control (Control) in the experiment, while camptothecin-treated (2 µM) cells were treated as the positive control. Additionally, for each concentration, a blank sample constituting a solution of BCI and culture medium without cells was included. After exposure, plates were removed from the incubator to bring the medium to room temperature (RT), and then 100 μL of Caspase-Glo^®^ 3/7 reagent was added for 1 h. A SpectraMAX i3 Molecular Devices reader was used to measure luminescence.

### 2.5. Flow Cytometry Cell Cycle Distribution Analysis

This method uses propidium iodide to detect DNA, the amount of which changes during different phases in the cell cycle. For this purpose, cells were cultured under controlled conditions (5% CO_2_, 37 °C) in 6-well plates for 24 h (the density is provided in Table 1. Subsequently, they were exposed to 2 and 4 µM of BCI compound for another 24 h. After incubation, the cells were detached using trypsin and transferred to conical tubes, followed by PBS washing. The cells were then centrifuged (10 min, 4 °C, 1400 rpm), fixed with 70% cold ethanol, and washed with PBS. Subsequently, the cells were treated with RNase A Solution (10 u/mL) and stained with propidium iodide (5 μg/mL) for 30 min at 37 °C in the absence of light. Flow cytometry measurements were conducted using a Becton Dickinson LSR II instrument at a wavelength of 530 nm. The resulting data, consisting of 10,000 events per sample, were examined using FlowJo 10.8.1 software.

### 2.6. Analysis of BCI Modulation Capacity

DLD1 cells were selected for further studies due to the highest cytotoxicity of BCI in these cells and the observed increase in caspase 3/7 levels. The investigation into the influence of BCI on the activity of irinotecan and oxaliplatin began by determining the IC_50_ values of each compound in monotherapy. For this purpose, the MTT test was performed as described above (Section 2.3), and the cells were treated with the compounds for 72 h.

#### 2.6.1. Impact BCI on the Cytotoxic Effect of Drugs

The viability of DLD1 cells was evaluated following treatment with irinotecan or oxaliplatin at three concentrations: 0.25 IC_50_, 0.5 IC_50_, and IC_50_. These treatments were administered both individually and in combination with BCI at concentrations of 0.25 IC_50_ and 0.5 IC_50_, and with KU60019 at a concentration of 0.5 IC_50_. The last two groups consisted of cells treated with the drugs and BCI (0.25 IC_50_ and 0.5 IC_50_) and KU60019 together. Additionally, one group consisted of cells not treated with any compound (negative control) and treated only by particular concentrations of BCI and KU, and BCI and KU together, without the drug having been taken into consideration. Incubations were carried out for 72 h, after which cell viability was analyzed. 

#### 2.6.2. Flow Cytometry Analysis of Apoptosis

During the apoptosis, membrane lipid transfer occurs, resulting in a shift of phosphatidylserine (PS) from the inner to the outer layer of the cell membrane. Annexin V can capture PS and combining it with a fluorescent dye allows cytometric detection of this complex [15,16].

Detection of externalized PS in DLD1 cells was conducted by using Alexa Fluor^®^ 488 Annexin V/Dead Cell Apoptosis Kit according to the manufacturer’s recommendations. Cells were seeded in 6-well plates (density as indicated in Table 1 for a 24 h accommodation period (standard conditions: 37 °C, 5% CO_2_). Test substances (BCI, oxaliplatin, irinotecan, and KU60019) at a predetermined concentration equal to the IC_50_ value (after 72 h) and combinations were added to the cells for 24 h incubation. The cells were subsequently trypsinized, and the entire contents of the wells were transferred to flow cytometry tubes. The tubes were then centrifuged at 1400 rpm for 10 min at 4 °C. The resulting cell pellet was washed with cold PBS and centrifuged again. Afterward, the cells were stained according to the manufacturer’s protocol. Flow cytometry measurements were carried out using a Becton Dickinson LSR II instrument at a wavelength of 530 nm. The resulting data, which comprised 10,000 events per sample, were analyzed using FlowJo 10.8.1 software.

#### 2.6.3. Synergy/Antagonism Investigation

Assessment of the type of interaction between the compounds was conducted by the MTT test (Section 2.3) and analyzed using Combenefit (https://www.cruk.cam.ac.uk/research-groups/jodrell-group/combenefiT, accessed on 12 June 2023) [17] and SynergyFinder 3.0 software (https://synergyfinder.fimm.fi, accessed on 12 June 2023). These programs quantify the level of synergy or antagonism in the combination by comparing the observed response of the drug combination to the expected response. The expected response is calculated using a reference model that assumes no interaction between the drugs. Popular reference models include the highest single agent (HSA), Bliss, Loewe, and zero interaction power (ZIP) models. 

To test the interactions between the compounds, DLD1 cells were treated by two-fold serial dilution of either BCI (concentration range 0.3–4.8 µM) in combination with irinotecan (concentration range 1.63–26.08 µM), oxaliplatin (concentration range 1.15–18.4 µM), or KU60019 (concentration range 1.63–26.12 µM) for 72 h. After the incubation period, the test was continued as described above. The test was conducted in four repetitions.

### 2.7. Statistical Analysis

Each experiment was conducted at least three times. Survival curves were generated using GraphPad Prism9 software (San Diego, CA, USA). This same program was used for the statistical analysis of collected data. The Shapiro–Wilk test was used to examine the normality of the distribution. The ANOVA post hoc tests including Dunnett’s test and Tukey’s test were used for the comparison of means between more than two groups while the Kruskal–Wallis test was used to compare medians. A significant difference was considered when the *p*-value was less than 0.05 (*p* < 0.05).

### 2.8. Computational Studies

#### 2.8.1. Drug Likeness and ADMET

The pharmacokinetic characteristics of the BCI were predicted using pkCSM (https://biosig.lab.uq.edu.au/pkcsm/ (accessed on 15 January 2023)) [18], SwissADME (http://www.swissadme.ch/ (accessed on 15 January 2023)) [19], and PreADMET (https://preadmet.webservice.bmdrc.org/ (accessed on 15 January 2023)) online servers (accessed on 15 January 2023) by including the canonical SMILES string obtained from PubChem (https://pubchem.ncbi.nlm.nih.gov/compound/BCI-hydrochloride (accessed on 15 January 2023)).

#### 2.8.2. Target Prediction

The key macromolecular targets that are supposed to interact with BCI were predicted by the SwissTargetPrediction (http://www.swisstargetprediction.ch/ (accessed on15 January 2023)) and the SuperPred webservers (https://prediction.charite.de/subpages/target_prediction.php (accessed on 15 January 2023)). Additionally, structures of DUSP-2, -5, -6, -7, -8, -13, -14, -18, and -28 were included in the studies as probable molecular targets based on existing literature [14,20].

#### 2.8.3. Molecular Docking

With the intent to establish the probable mechanism of action for the BCI, it has been computationally screened against various macromolecular targets. Molecular docking was performed for targets with a known PDB structure (https://www.rcsb.org/ (accessed on 22 January 2023)). The energy-minimized three-dimensional structure of BCI was procured from the NCBI PubChem database. The prepared ligand was computationally screened against calcium sensing receptor (CASR) (PDB ID: 7dtt) [21], C-X-C chemokine receptor type 3 (CXCR3) (PDB ID: 6wzk) [22], cyclin-dependent kinase-5 (CDK5) (PDB ID: 1unl) [23], cathepsin D (CPSD) (PDB ID: 4od9) [24], casein kinase II subunit alpha (CK2A1) (PDB ID: 6tls) [25], cyclin-dependent kinase-1 (CDK1) (PDB ID: 6gu2) [26], dopamine D1 receptor (DRD1) (PDB ID: 7ckx) [27], dual specificity phosphatase 13a (DUSP13) (PDB ID: 5xjv) [28], dual specificity phosphatase 14 (DUSP14) (PDB ID: 2wgp) [29], dual specificity phosphatase 18 (DUSP18) (PDB ID: 2esb) [30], dual specificity phosphatase 2 (DUSP2) (PDB ID: 1m3g) [31], dual specificity phosphatase 28 (DUSP28) (PDB ID: 5y15) [32], dual specificity phosphatase 5 (DUSP5) (PDB ID: 2g6z) [33], dual specificity phosphatase 6 (DUSP6) (PDB ID: 1hzm) [34], dual specificity phosphatase 7 (DUPS7) (PDB ID: 4y2e) [35], dual specificity phosphatase 8 (DUSP8) (PDB ID: 4jmk) [36], dipeptidyl peptidase IV (DPP4) (PDB ID: 4pv7) [37], glucagon-like peptide-1 receptor (GLP-1R) (PDB ID: 5vai) [38], human apurinic/apyrimidinic endonuclease 1 (APE1) (PDB ID: 6bow) [39], human indoleamine 2,3-dioxygenase-1 (IDO1) (PDB ID: 6e43) [40], human topoisomerase-II beta (TOP2β) (PDB ID: 3qx3) [41], intercellular adhesion molecule (ICAM-1) (PDB ID: 5mza) [42], lysosomal pro-X carboxypeptidase (PRCP) (PDB ID: 3n2z) [43], Mu opioid receptor (OPRM1) (PDB ID: 4dkl) [44], plasminogen activator inhibitor type-1 (PAI-1) (PDB ID: 3cvm) [45], rho-associated protein kinase-1 (ROCK1) (PDB ID: 3v8s) [46], rho-associated protein kinase-2 (ROCK2) (PDB ID: 6ed6) [47], serotonin 5a (5-HT5a) receptor (PDB ID: 7x5h) [48], signal transducer and activator of transcription-3 (STAT3) (PDB ID: 6nuq) [49], somatostatin receptor 3 (SSTR3) (PDB ID: 7xms) [50], tripartite motif-containing 24 (TRIM24) (PDB ID: 4ybm) [51], tyrosine-protein kinase JAK1 (PDB ID: 6dbn) [52], tyrosine-protein kinase JAK2 (PDB ID: 4c61) [53], tyrosine-protein kinase JAK3 (PDB ID: 4hvd) [54], tyrosine-protein kinase TYK2 (PDB ID: 6dbm) [52].

For the purpose of validating the molecular docking simulation, the structures of all the macromolecular targets were first docked versus their source ligand, which had been previously complexed in the fetched PDB structure to determine its affinity toward the particular protein, followed by docking with the BCI ligand. 

Hydrogens were added, redundant water molecules were removed, Gasteiger charges were calculated, and atom types were assigned in Autodock-4 (AD4) before the downloaded macromolecular targets were saved in the standard Autodock format, PDBQT.

The flexibility was assigned in the ligand BCI followed by saving its structure in the Autodock format. For all the molecular targets considered here, an appropriate grid box was constructed to include all the extended conformations of the complexed reference ligands and the vast majority of the interacting residues. Each projected target had its own grid parameter file (GPF) containing the grid parameters that were used by the Autogrid utility in the Autodock suite to create the map files needed for running molecular docking simulations. Table 2 lists the grid parameters for all of the study’s targets.

Autodock was used to run molecular docking simulations utilizing the map files for different atom types of the macromolecular target and ligand that were generated by the Autogrid tool.

#### 2.8.4. Molecular Dynamics (MD) Simulation

Molecular dynamics simulations were used to verify the acquired docking results for the temporal stability of the macromolecular drug-receptor intricate interactions. The macromolecular complex of ligand BCI against TRIM24, CK2A1, and CDK5 was shortlisted for molecular dynamics on the basis of the best docking score. Desmond software (version 2019.4) with an OPLS force field was used to run simulations for 100 ns duration at a constant temperature and pressure. Desmond utilities were used to perform a variety of trajectory analyses, including measuring root-mean-square deviation (RMSD), root-mean-square fluctuation (RMSF), and interacting bonds [55,56].

#### 2.8.5. Prime MM-GBSA Analysis

The binding affinity of BCI with CDK5, TRIM23, and CK2A1 was estimated using the Prime Molecular Mechanics Generalized Born Surface Area (MM-GBSA) module of Schrödinger (Prime, Schrödinger, LLC, NY, USA). The optimization feature embedded in the Prime module was used to minimize the protein–ligand complex and further minimization was carried out using Optimized Potential for Liquid Simulation—All Atom (OPLS-AA) force field. The following formula was used to calculate the free energy of binding for the protein–ligand complex:

ΔG bind = G complex − (G protein + G ligand) G = EMM + GSGB + GNP

The energies of the complex, protein, and unbound ligand were represented as G complex, G protein, and G ligand, respectively. The molecular mechanics energies (EMM), in addition to SGB polar solvation model (GSGB) and nonpolar solvation (GNP), were together represented as G.

## 3. Results

### 3.1. BCI Cytotoxicity

MTT assay was conducted to estimate BCI cytotoxicity on three different colon cancer cell lines. Two incubation times were used to observe the dynamics of changes in cell viability.

**Figure 2:**
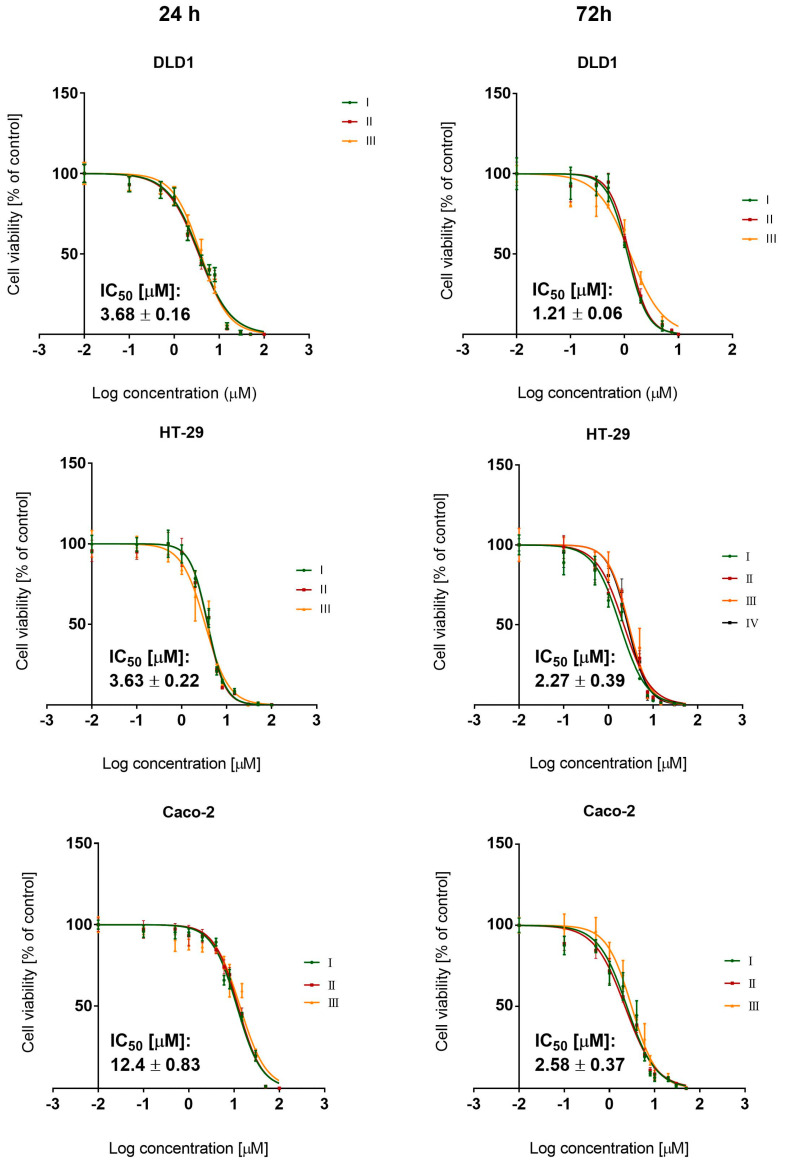
Dose–response curves (representing relative cell viability) obtained for 24 and 72 h exposure of colon cancer cells (DLD1, HT-29, and Caco-2 lines) to BCI. The results were provided with the mean IC_50_ value calculated from at least three independent experiments (±SD).

Results obtained from the MTT test showed that the DUSP6 inhibitor exhibited cytotoxic activity toward all examined cell lines. BCI had a dose-dependent ability to reduce the viability of colon cancer cells in micromolar concentrations (mean IC_50_ value range: 1–12 μM). Dose–response curves demonstrated cytotoxic effects depending on the exposure time. The extension of the incubation time to 72 h resulted in IC_50_ values that were 1.5–4 times lower than those observed for 24 h incubation (Figure 2). Increasing the incubation time also nullified the initially marked differences in the cellular response to the test compound between cell lines. However, DLD1 cells proved to be the most sensitive to BCI at both time points.

### 3.2. Study on Caspase 3/7 Activity

**Figure 3:**
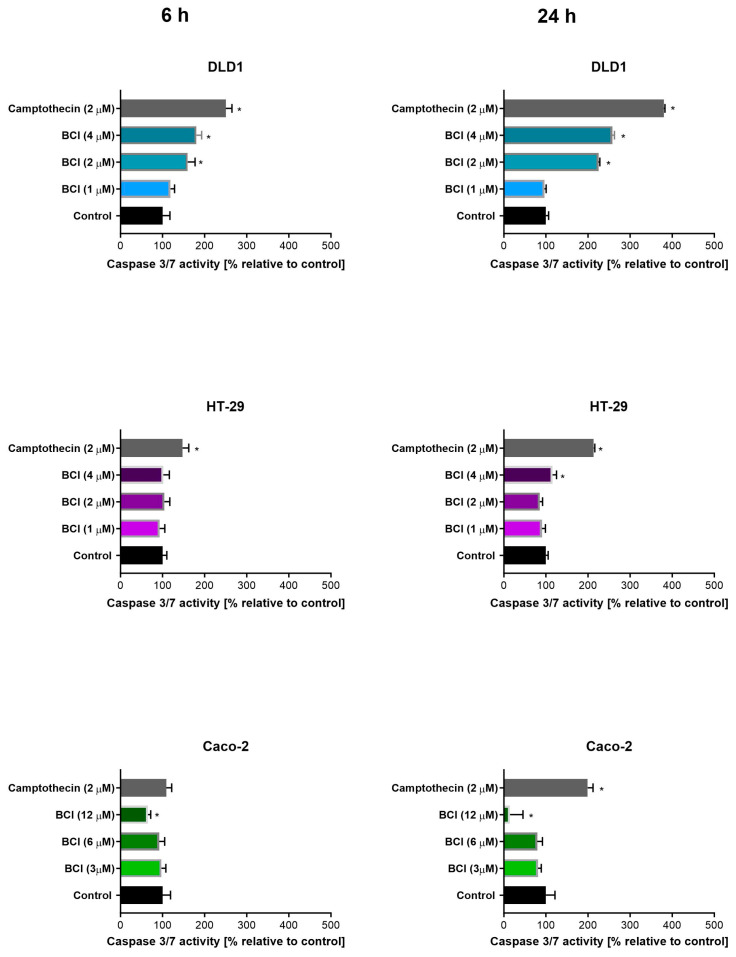
Determination of caspase 3/7 activity in colon cancer cells after 6 and 24 h exposure to BCI compound in comparison to untreated cells. Data are presented as percentages relative to negative control defined as 100% (±SD); 2 µM of camptothecin was used as the positive control of the assay. * indicates statistical significance compared to negative control (*p* < 0.05).

Examination of caspase 3/7 activity indicated dose-dependent induction of apoptosis in cells of the DLD1 and HT-29 lines in the presence of a DUSP6 inhibitor (Figure 3). An increase in enzyme activity was observed after 24 h exposure of DLD1 cells to BCI used in both tested concentrations, while for HT-29 a similar effect was observed only for the highest concentration of the inhibitor. A shorter exposition (6 h) of DLD1 to the tested compound resulted in an increase in luminescence only at the highest concentration investigated. In Caco-2 cells, the highest tested concentration of BCI reduced luminescence intensity at both time points. 

### 3.3. Cell Cycle Analysis

**Figure 4:**
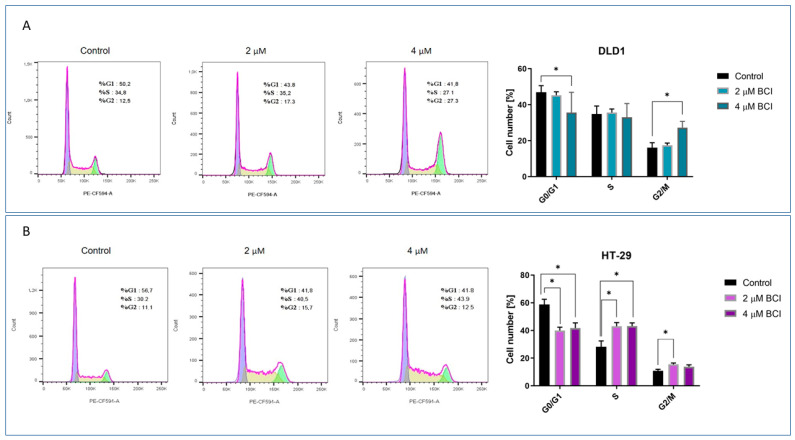
The sample histograms illustrate changes in the cell cycle distribution of DLD1 (**A**) and HT-29 (**B**) cells after 24 h exposition to the BCI compound. The data are presented as the mean percentage of cells (±SD) in each phase of the cell cycle (G1—blue, S phase—yellow, G2/M—green color) in the control group and the groups exposed to 2 and 4 μM BCI. * indicates statistical significance compared to negative control (*p* < 0.05).

Cell cycle analysis was performed in DLD1 and HT-29 cells, given the increased caspase enzymatic activity following their 24 h incubation with BCI. The example histograms (Figure 4) represent the percentage distribution of cancer cells in different phases of the cell cycle. Results showed that the tested concentrations of BCI impacted the progression of the cell cycle. DLD1 cells exhibited an elevated percentage of cells in the G2/M phase (from 16.17% to 27.27%) with a reduced population of cells in the G1/G0 phase (from 46.92% to 35.63%) after exposure to 4 µM BCI. Conversely, in HT-29 cells, the population of cells in the G1/G0 phase decreased (from 58.78% to 39.98 for 2 µM and 41.78% for 4 µM), with a concomitant increase in the population of cells in the S phase (from 28.25% to 43% and 43.2%, respectively) after treatment with BCI in both tested concentrations. A slight increase in the population of cells in the G2/M phase was observed for BCI used at the concentration of 2 μM (from 10.83% to 15.5%).

### 3.4. Modulation of Chemotherapeutic Agent Activity

#### 3.4.1. Cytotoxicity Study

**Figure 5:**
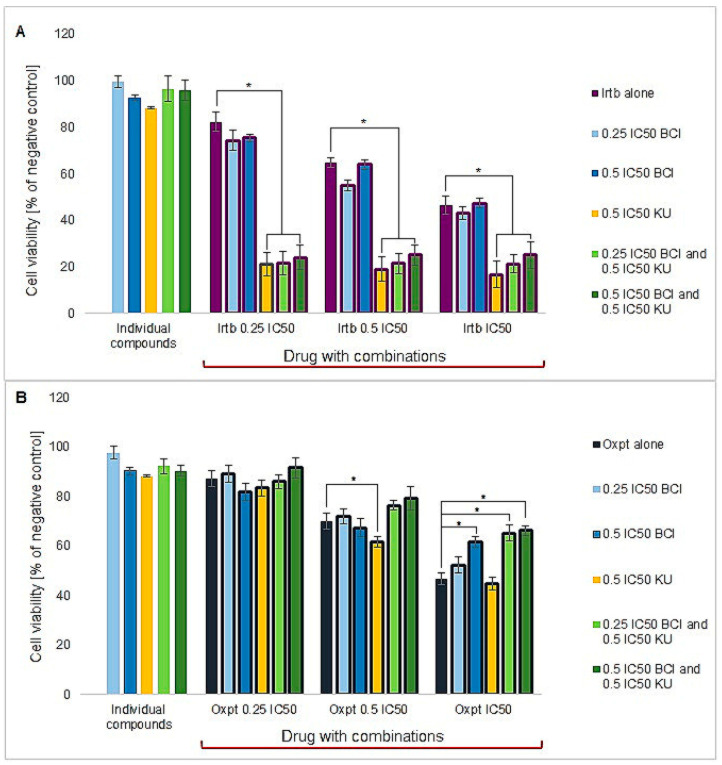
The viability of DLD1 cells following 72 h exposure to investigated compounds: (**A**) irinotecan and (**B**) oxaliplatin and their combinations with BCI and KU60019 (KU). Data are presented as the mean percentage (±SEM) of viable cells with respect to the negative control defined as 100%. * indicates statistical significance compared to individual drug concentrations (*p* < 0.05).

The cytotoxicity of chemotherapeutic agents such as irinotecan and oxaliplatin in monotherapy and combination with either BCI or KU60019 (KU) and combination with both BCI and KU was examined by MTT test. The data obtained indicated that in DLD1 cells, BCI presented more than five times higher cytotoxicity than the chemotherapeutic drugs investigated (Table 3). The results revealed that the tested modulatory concentrations of BCI and KU did not induce a decrease in the viability of colon cancer cells to the point below 85% (Figure 5). BCI alone did not exhibit any effect on irinotecan cytotoxicity. However, a significant decrease in cell viability was observed when BCI was combined with an ATM inhibitor. Nonetheless, the cytotoxic effect was comparable to that achieved when the drug was used in conjunction with KU alone. Regarding oxaliplatin combinations, BCI had a detrimental effect on the efficacy of this chemotherapeutic agent in colon cancer cells. The results showed an increase in cell viability when BCI was used at higher concentrations in combination with oxaliplatin at IC_50_ concentration, and when combined with an ATM inhibitor (both concentrations of BCI).

#### 3.4.2. Apoptosis Detection

**Figure 6:**
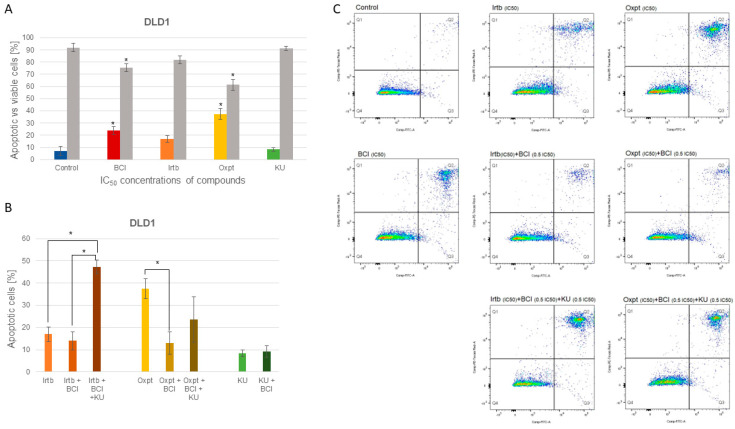
Apoptosis induction in DLD1 cells: (**A**) displays the mean percentage (±SD) of apoptotic and viable cells following 24 h incubation with the tested compounds at concentrations equal to their respective IC_50_ values; (**B**) illustrates the mean percentage (±SD) of apoptotic cells after the exposure of DLD1 cells to either IC_50_ concentration of irinotecan or IC_50_ concentration of oxaliplatin in monotherapy and in combination with 0.5 IC_50_ BCI or BCI and 0.5 IC_50_ KU, and KU and KU with BCI; (**C**) shows the distribution of DLD1 cells classified as necrotic (Q1), early apoptotic (Q4), late apoptotic (Q2), and viable cells (Q3). Additional histograms are presented in Figure S1 in the Supplementary Materials. * indicates statistical significance compared to negative control (*p* < 0.05).

The phosphatidylserine detection studies revealed that BCI at a concentration of 1.2 µM (after 24 h incubation of cells) induced an increase in the population of apoptotic cells, which exceeded 20% compared to the control. Higher values were only achieved when cells were treated with oxaliplatin. In the study of the combination of BCI (at a concentration equal to 0.5 IC_50_ value) with chemotherapeutic agents, no influence on the activity of irinotecan was observed. The addition of KU60019 resulted in the enhanced antitumor effect of the chemotherapeutic drug. In the case of oxaliplatin, a significant decrease in the population of apoptotic cells was observed upon the addition of BCI compared to the activity of the drug used as monotherapy (Figure 6).

#### 3.4.3. Synergy Studies

**Figure 7:**
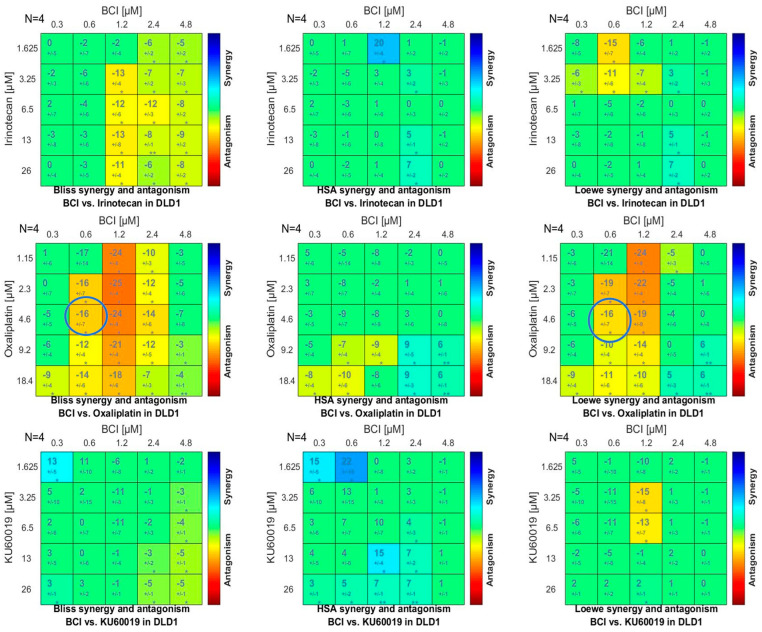
The synergy distribution matrix obtained by applying Combenefit software. The phenotypic response of DLD1 cells is expressed chromatically, where synergistic interaction is represented by blue and antagonistic interaction is represented by red. Circles indicate the most antagonistic concentration combinations used in previous experiments (Section 3.4.1 and Section 3.4.2). Data were obtained from four independent experiments (*n* = 4). * *p* < 0.05; ** *p* < 0.01.

**Figure 8:**
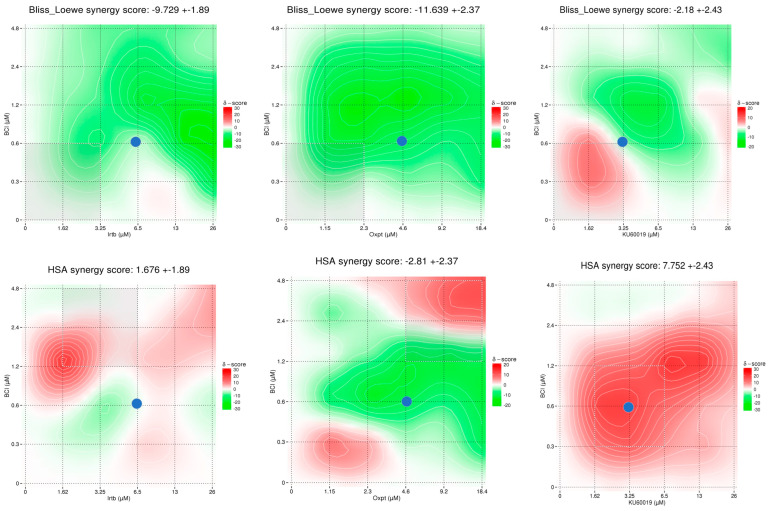
Drug combination data analysis from the SynergyFinder 3.0 program. The phenotypic response of DLD1 cells is expressed chromatically, where synergistic interaction is represented by red and antagonistic interaction is represented by green. The color spectrum is numerically expressed within the range 20 to −20. Data were obtained from four independent experiments (*n* = 4). The blue dots represent concentrations used in previous experiments.

Analysis of interaction between BCI and the examined chemotherapeutics and ATM inhibitor confirmed the results obtained in previous experiments. Both the Combenefit and SynergyFinder 3.0 software indicated antagonism in the action of BCI and oxaliplatin (δ score from −9 to −16 depending on the selected model). The concentrations of irinotecan and KU60019 tested in earlier experiments showed neither synergy (>10) nor antagonism (<−10) in combination with BCI, meaning that the interaction is likely to be additive or there is no interaction between them (from −10 to 10). Some synergy scoring models indicated that BCI and ATM inhibitor have the potency to be synergic in low concentrations (Figure 7 and Figure 8). However, the synergistic effect of the previously tested combination was only confirmed by the HSA model introduced in SynergyFinder 3.0 software, while the main reference models Bliss and Loewe only indicate an additive effect.

### 3.5. Computational Analysis

#### 3.5.1. Drug Likeness and ADMET

Drug discovery is a long and complex process that can be broken down into several overall steps, including the identification of disease targets, the validation of those targets, the high-throughput identification of “hits” and “leads”, the optimization of leads, and the preclinical and clinical evaluation of the selected structures. Each of these steps constitutes an enormous field of study on its own. However, except the first two phases, all of the subsequent stages entail the characterization of the absorption, distribution, metabolism, and excretion (ADME) and toxicity (T) of the compounds that are being sought as prospective drug candidates. It is believed that insufficient ADMET properties are to blame for the clinical failures of approximately fifty percent of all Investigational New Drug (IND) filings. As a result, it should come as no surprise that the pharmaceutical sector is searching for any possible means to reduce this attrition in the current context, which is characterized by social and governmental pressure on the expenses of healthcare. The creation of mathematical models, also known as in silico screens, to accurately predict ADMET properties based purely on the molecular structure is at the core of this attempt to save costs and shorten the length of development cycles [57,58,59]. Lipinski’s rule of five has become standard for the estimation of the drug-likeness of the compounds. According to this rule, poorly bioavailable drugs exceed the molecular weight > 500, Clog P > 5, hydrogen-bond donor > 5, and hydrogen-bond acceptor > 10 in the structure [60]. However, the Ghose, Veber, and Edgan models can also be used.

ADMET and druglikeness characteristics of BCI predicted using the SwissADME tool (http://www.swissadme.ch/ (accessed on 15 January 2023)) are presented in Table 4.

**BCI** was predicted to follow the Lipinski, Ghose, Veber, Egan rules with no violations. Simultaneously, the pharmacokinetics characteristics of the BCI were predicted using pkCSM (https://biosig.lab.uq.edu.au/pkcsm/ (accessed on 15 January 2023)) [18], SwissADME (http://www.swissadme.ch/ (accessed on 15 January 2023)) [19], and PreADMET (https://preadmet.webservice.bmdrc.org/ (accessed on 15 January 2023)) online servers accessed on 15 January 2023 (Table 5).

BCI showed high predicted gastrointestinal (GI) absorption with 94.7% of the drug absorbed in the GI tract, conferring its possible oral bioavailability. Ambiguous results were obtained when predicting BCI’s cytochrome P450 (CYP) inhibition. CYPs are involved in the occurrence of potential side effects following drug administration and may strongly impact the bioavailability of the compounds. Therefore, the BCI inhibitory effects on CYPs should be closely investigated [61]. The objective of the Bioavailability Score is to predict the likelihood that a substance will have an oral bioavailability in rats of at least 10%. BCI has been shown to exhibit a bioavailability score of 0.55 (55%) [62].

BCI showed good blood–brain barrier (BBB) permeability facilitating its accumulation in the central nervous system (CNS) compartment, but the activity may be restricted as it may interact with P-glycoprotein [63]. Adenosine triphosphate (ATP)-binding cassette (ABC) superfamily protein members (including P-glycoprotein) constitute pivotal transporters influencing drug efflux that is highly expressed in tumor cells. Therefore, the BCI interaction with P-glycoprotein may limit its antitumor efficacy [64]. 

The mutagenicity of drugs is an important endpoint that restricts the implementation of the potential molecule into the clinic. The Ames test constitutes a gold standard of mutagenicity assessment in early drug development. BCI exhibited no Ames toxicity as predicted by the pkCSM software [65].

Cancer patients are at an increased risk of experiencing unfavorable cardiovascular events as a result of the malignant process itself or the treatment for the disease. Late effects of cancer treatment can become clinically visible decades after therapy has been completed. Therefore, it is crucial to predict the cardiotoxic potential of drug-like molecules. Cardiotoxicity may result from the inhibition of the potassium ion channel of the human ether-a-go-go-related gene (hERG), which can lead to the occurrence of long QT syndrome (LQTS) and heart failure [66]. In the in silico prediction with the PreADMET server, BCI was found to have a medium risk of triggering cardiotoxic effects.

The liver is the major organ of drug detoxification. Therefore, hepatotoxicity constitutes an obstacle to the introduction of drugs into clinical practice [67]. BCI was found to possess hepatotoxic properties in the in silico prediction using the pkCSM webserver. 

Synthetic accessibility (SA) is a measure used to assess the ease or difficulty of synthesizing a given molecule. In the context of this description, SA is determined using a fragmental method based on the analysis of a vast collection of more than 13 million compounds that can be readily obtained from vendors. The method works by breaking down each molecule into smaller molecular fragments called “FP2 bits”, which are essentially specific chemical building blocks or substructures. The frequency of occurrence of these fragments in the large collection of compounds is then analyzed. The key idea is that if a molecular fragment is common among the compounds, it suggests that it is relatively easy to synthesize because it is readily available from vendors or accessible through standard chemical reactions. On the other hand, rare fragments indicate that they may be challenging or expensive to synthesize because they are not readily commercially available or require complex synthetic pathways.

To calculate the SA Score for a particular molecule, the fragmental contributions to synthetic accessibility are summed, and additional terms describing the molecule’s size and complexity are incorporated to refine the assessment. This normalization step ensures that the SA Score falls within a specific range, which is from 1 (indicating very easy synthesis) to 10 (representing very difficult synthesis). BCI showed SA of 3.58, indicating ease of synthesis [19].

#### 3.5.2. Target Prediction

In addition to the good ADMET profile, a potential drug candidate should demonstrate adequate efficacy against the specifically determined therapeutic target or targets. The knowledge about the chemical structure of the chemical compound can be used for the prediction of the molecular targets of the promising chemical entity [68]. The key macromolecular targets which are supposed to interact with BCI were predicted by the SwissTargetPrediction (http://www.swisstargetprediction.ch/ (accessed on 15 January 2023)) and the SuperPred webservers (https://prediction.charite.de/subpages/target_prediction.php (accessed on 15 January 2023)). Fifteen targets with the highest probability, predicted using SwissTargetPrediction, were subjected to molecular docking, and SuperPred webserver targets with model probability and accuracy of at least 80% were included for molecular docking studies, as described below. Additionally, we performed docking studies of BCI toward dual-specificity phosphatase (DUSP) enzymes indicated as primary targets of the BCI, based on literature data [14,20].

#### 3.5.3. Molecular Docking

Molecular docking of the BCI with the anticipated targets demonstrated that the BCI has strong interactions with cyclin-dependent kinase 5 (CDK5), casein kinase II subunit alpha (CK2A1), and tripartite motif-containing 24 (TRIM24) (the assumption was based on the observed binding score). Table 6 displays the binding score for BCI along with all of the reference ligands for each of the macromolecular targets.

#### 3.5.4. Molecular Dynamics (MD) Simulation

**Figure 9:**
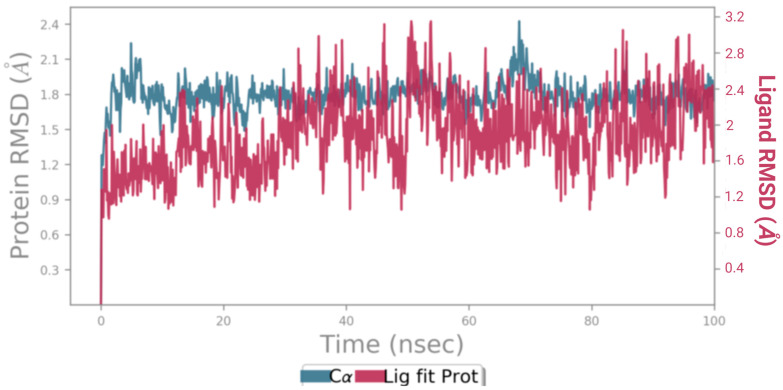
Root-mean-square deviation (RMSD) trajectory for the complex ligand BCI and the macromolecular backbone of cyclin-dependent kinase 5 (CDK5) showing stable behavior with a slight fluctuation, as determined by MD simulation of 100 ns.

The macromolecular complexes of BCI and the shortlisted macromolecular targets CDK5, CK2A1, and TRIM24 were further evaluated for thermodynamic stability with time by executing an MD simulation of 100 ns. MD simulation analysis revealed that the macromolecular complex of BCI against CDK5 exhibited the highest stability and the lowest fluctuation throughout the simulation process. Root-mean-square deviation (RMSD) was used to assess the stability and structural changes to the protein backbone. With an average RMSD value of 1.5–2.1 for the macromolecular backbone and 1.5–2.8 for BCI, the trajectories acquired from the MD simulations of BCI-CDK5 complexes have shown their stability throughout the 100 ns simulation, as shown in Figure 9.

To determine how much the amino acids shifted from their starting positions, we used a calculation called root-mean-square fluctuation (RMSF). This calculation focused on the movement of specific atoms within the macromolecules. We observed that changes in the active site were inversely related to the movement of amino acids, meaning when one increased, the other decreased. The RMSF graph displayed amino acid positions on the horizontal axis and their corresponding RMSF values on the vertical axis. The RMSF values for the core structure of these large molecules ranged from 0.5 to 2.0 angstroms. Our calculations showed that the RMSF values for the complexed system were lower for the active amino acids, with an average change in the ligand BCI falling within the range 0.8 to 1.2 angstroms. This demonstrated that the fluctuations within the active site of the macromolecules were within acceptable limits.

**Figure 10:**
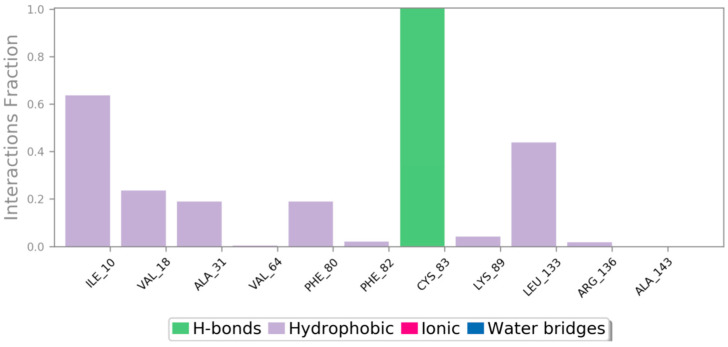
The specific interactions between macromolecular complexes that were seen during the 100 ns MD simulation. Hydrogen bonds are represented by green bars and hydrophobic interactions by purple bars.

Throughout the entire simulation, we conducted an analysis of the secondary structure (SSE) of the macromolecules. This analysis revealed that approximately 40.62% of the structure consisted of SSE. Within this percentage, about 25.28% constituted the alpha-helices, and 15.34% beta-sheets. These structural elements appeared to remain relatively stable during the simulation. The stability of the protein–ligand complexes was attributed to the formation of hydrogen bonds, hydrophobic interactions, and ionic attractions that occurred throughout the entire molecular dynamics (MD) simulation. To assess the stability of the ligand BCI, we closely monitored the strength of these interactions over the simulation period. Our analysis of the ligand BCI’s interaction with CDK5 during the simulation indicated that the ligand had hydrophobic interactions with amino acids Ile10, Val18, Ala31, Phe80, Phe82, Lys89, Leu133, and Arg136. Additionally, amino acid Cy83 formed a hydrogen bond with the ligand BCI. The ligand interaction observed between the ligand BCI and macromolecular target CDK5 is demonstrated in Figure 10.

#### 3.5.5. Prime MM-GBSA Analysis

The prime MM-GBSA calculation was performed to estimate the binding affinity of BCL inside the active site of CDK5, TRIM23, and CK2A1. The calculated binding free energy was found to be −57.4, −59.23, and −22.62 kcal/mol for CDK5, TRIM23, CK2A1, respectively, and BCI complexes (Table 7). Based on the observation, it was found that BCI shows a stronger binding affinity with CDK5 and TRIM24 than CK2A1. In addition, other binding free energy parameters such as coulomb energy, van der Waals, covalent energy, and nonpolar solvation terms were found higher for both CDK5 and TRIM24 complexes, indicating the favorable binding of BCI with CDK5 and TRIM24. The results confirm the contribution of various binding free energy parameters in optimizing the protein–ligand complexes.

## 4. Discussion

Conducting in silico ADMET screens of chemical compounds of interest is crucial for early-stage drug discovery and development. It helps identify potential safety and efficacy issues in a cost-effective and time-efficient manner, allowing researchers to prioritize and optimize lead compounds. By predicting the pharmacokinetic and toxicological properties of the compounds, in silico screens reduce the number of experimental iterations required and contribute to the selection of safer and more promising drug candidates for further investigation [57]. BCI showed drug-likeness properties and demonstrated high predicted GI absorption, with 94.7% of the drug being absorbed in the GI system, indicating oral bioavailability. However, when predicting BCI’s cytochrome P450 inhibition, ambiguous findings were found, which could impair drug bioavailability. BCI exhibited high permeability across the blood–brain barrier, allowing it to accumulate in the central nervous system compartment. Its effectiveness, however, may be limited by its interaction with P-glycoprotein limiting its antitumor effectiveness. Furthermore, cancer patients are more likely to experience unfavorable toxicities. According to in silico predictions, BCI has no risk for Ames test mutagenicity, a medium risk in causing cardiotoxic effects, and a high risk in causing hepatotoxicity. Liver damage is commonly caused by drugs and hepatotoxicity is one of the most common side effects of medicinal chemical agents. Although practically any clinical pathological pattern of acute or chronic liver illness can occur, acute hepatitis and/or cholestasis are the most common clinical presentations. The parent drug or metabolites that either directly influence cell biochemistry or provoke an immunological response are typically involved in the pathophysiology of drug-induced liver disease. In terms of damage pattern and delay, each hepatotoxin has its unique signature [69]. Hepatotoxicity seldom stops preclinical drug development. Liver toxicity is usually reversible and may be tracked in people using sensitive serum enzyme tests, unlike other target organ damage. Thus, a drug found to be hepatotoxic in animals is often tested in humans to determine its hepatotoxic properties. If a drug has high therapeutic potential, liver damage can be tolerable and manageable with complementary modalities [67]. 

Cancer is a complex and heterogeneous disease, often involving multiple signaling pathways and molecular abnormalities. By simultaneously targeting multiple relevant targets, it may be possible to enhance therapeutic efficacy, overcome resistance mechanisms, and potentially achieve synergistic effects. However, the development of effective multitargeting therapeutics requires careful consideration of the specific cancer type, the interplay between targets, potential side effects, and the overall treatment strategy. Targeting multiple molecular targets involved in cancer development with a single agent can have potential drawbacks and challenges. One concern is the increased risk of toxicity and adverse effects due to the broader impact on multiple biological processes. Additionally, targeting multiple targets simultaneously may lead to unwanted interactions, such as antagonistic effects between different pathways or incomplete inhibition of specific targets, which could compromise therapeutic efficacy. We have therefore performed in silico screening for the predicted molecular targets of BCI and DUSP enzymes that have been established previously as primary targets of the compound [14,20]. We found that three targets exhibited significant stability with the compound. These include CDK5, CK2A1, and TRIM24. Nevertheless, the greatest stability was shown for the complex of BCI and CDK5.

The most notable pathophysiological role of CDK5 was shown in Alzheimer’s disease. Overactivation of CDK5 is linked to tau hyperphosphorylation, which leads to the production of neurofibrillary tangles [70,71]. Given the ADMET properties of BCI, it could constitute a promising lead compound for the development of selective CDK5 inhibitors in this neurodegenerative disease. Here, however, we focused on targeting CDK5 in the context of cancer cells. The kinase is involved in tumor proliferation, migration, and angiogenesis, and may confer resistance to chemotherapy and affect antitumor immunity. CDK5 inhibition or knock-down has been shown to have anticancer properties via multiple pathways and can enhance the killing effect of chemotherapeutics. CDK5 participates in the DNA damage response (DDR) primarily by phosphorylating key DNA damage response proteins such as ATM kinase or apurinic/apyrimidinic endonuclease 1 (APE1), and suppression of this enzyme activity has been shown to regulate the DDR process and cancer progression as recently reviewed by multiple authors [72,73,74]. The inhibitory effects of BCI on CDK5 inhibition should be evaluated in future enzymatic assays.

In the current study, the cytotoxic and cytostatic effect of BCI on colon cancer cells, and BCI’s ability to modulate the activity of chemotherapeutics such as irinotecan and oxaliplatin, were examined. Moreover, ATM kinase inhibitor (KU60019 (KU)) was also included in the combination scheme. In the proposed in vitro model, the strong cytotoxicity of BCI was observed at micromolar concentrations (IC_50_ range 1.21–12.4 µM). However, variations in the sensitivity of cell lines to the BCI were noted. The DLD1 cells were most sensitive to the DUSP6 inhibitor in both investigated incubation times, while Caco-2 cells, which exhibit functional similarities to normal enterocytes, required a longer exposure to observe the inhibition of cell growth at a comparable level to the other tested cell lines. The decrease in cell viability associated with BCI activity has also been observed in several types of cancer cells. Neuroblastoma cells (KELLY, IMR-32, LAN-1, and SK-N-AS) where BCI exhibited EC_50_ (the half-maximal effective concentration) values in the range of concentrations from 0.42 to 1.34 µM. Significantly, the cell line with the strongest expression of DUSP6 turned out to be the least sensitive to the tested compound. Furthermore, DUSP6 knock-out subclones (KELD6-1, KELD6-2, and IMRD6-1) did not lose their susceptibility to BCI, and their EC_50_ values have not exceeded 0.5 µM [14]. In the study by James et al., the SKOV3 and OVCAR8 ovarian cancer cell lines exhibited a decrease in cell viability upon exposure to 3.75 μM BCI [75]. The dose and time-dependent decrease in the viability of cells upon treatment with BCI was also observed in lung cancer cells such as NCI-H1299, NCI-H460, and A549. Moreover, the cytotoxic effect was connected with the ability of cells to express TP53. NCI-H1299 *TP53*-defective cells were more sensitive to BCI treatment as expressed by both a decrease in proliferation and apoptosis [76]. In our study, the cytostatic effect was also observed in DLD1 and HT-29 cells. Cell cycle distribution analysis showed a strong reduction of cells in the G1/G0 phase and retention of cells in G2/M and, in the case of the HT-29 line, also in the S phase after 24 h exposure to BCI. In contrast, Shin et al. did not observe cell cycle alterations except for an increase in the subG1 phase. Interestingly, shRNA depletion of DUSP6 resulted in up to a 9 h delay in the S phase of epidermoid carcinoma A431 cells after thymidine synchronization [77].

Caspases 3/7 are the executive enzymes of the apoptosis process. Their activation is one of the initial steps in the pathway of programmed cell death. Our studies demonstrated that BCI, even at concentrations as low as 2 μM, triggers a notable increase in caspase 3/7 activity levels in DLD1 cells after just 6 h of incubation. After 24 h, the activity of these apoptotic enzymes was also demonstrated in HT-29 cells. In the case of Caco-2 cells, a decrease in caspase 3/7 activity was noted. However, the response to anticancer drugs can depend on the specific cancer type, stage, and microenvironment. These factors can influence the apoptotic response and caspase enzyme activity differently in different cancer types. The decrease in caspase enzyme activity in Caco-2 cells can be attributed to various reasons, some of which include off-target effects. Some anticancer drugs can have off-target effects, impacting other cellular processes that indirectly influence caspase activity. These off-target effects may lead to decreased caspase enzyme activity and reduced apoptosis. For example, other components of the apoptotic pathway downstream from caspases, such as Bcl-2 family proteins, may be affected. Activation of other response mechanisms such as autophagy or necrotic death may also be related to the observed decrease in apoptotic enzymes activity [78].

The induction of apoptosis in DLD1 cells was provided by flow cytometry phosphatidylserine detection. Twenty-four hour exposure of cells to 1.21 µM BCI resulted in the induction of apoptosis in more than 20% of cell populations. In their study, Shin et al. also observed typical apoptotic morphology of lung cancer cells and an increase in BAX expression with a concomitant decrease in BCL2 protein expression [76].

The exact mechanism of apoptosis induction by BCI remains unclear. Some studies suggest BCI’s contribution to excessive reactive oxygen species (ROS) generation [14,76]. It is known that the enzymatic activity of phosphatase could be inhibited by the oxidation of its cysteine residues in the catalytic site. Tumor necrosis factor α (TNFα)-mediated ROS generation can inhibit DUSP enzymes, so it is possible to induce that pathway by BCI especially since its cytotoxicity in pre-B acute lymphoblastic leukemia cells was decreased in the presence of free radical scavengers [14]. Another suggestion is given by Shien et al., who observed a drastic decrease in superoxide dismutase 2 (SOD2) and catalase mRNA with a concomitant increase in ROS levels in cells treated with BCI [76].

DUSP6 overexpression was confirmed in a variety of cancer types in both in vitro cells and surgical specimens from patients [79,80]. It has been shown that it may be related to cell resistance to chemotherapy [79,81,82]. The depletion of DUSP6 resulted in the sensitization of A431 cells to cytotoxic factors, including EGFR inhibitors, CPT11, dasatinib, and Aurora kinase inhibitors, without clear specificity toward any of them. Moreover, in in vivo conditions, the proliferation of subcutaneously implanted A431 xenograft tumor cells lacking DUSP6 was more restricted compared to cells with normal expression of this phosphatase after the action of EGFR inhibitors [77]. In the study with pharmacological DUSP6 inhibition, OVCAR8 and SKOV3 ovarian cancer cells showed greater sensitivity to carboplatin and paclitaxel in combination with BCI than to the drug alone. In both cases, the combined administration of 3.75 μM BCI and 100 μM carboplatin demonstrated a significantly enhanced cytotoxicity, surpassing the impact of either treatment alone (33.3% and 50.1% survival for OVCAR8 and SKOV3, respectively). This same concentration of BCI and 10 nM paclitaxel treatment resulted in a significantly higher rate of cell death, with survival rates of 25.4% in OVCAR8 and 45% in SKOV3 cells [75]. In a study by Wu et al., SGC7901/DDP gastric cancer cells resistant to cisplatin were sensitized to the drug in the presence of BCI via ERK activation. Additionally, in vivo experiments demonstrated that the administration of BCI amplifies the anticancer properties of cisplatin in xenografts derived from cells and patient-derived xenograft (PDX) models [10]. In our studies, we obtained contrasting results. In the case of combining BCI with oxaliplatin, a decrease in its anticancer activity was observed. Both MTT assay results and flow cytometer PS detection showed deterioration of oxaliplatin in the presence of BCI. Furthermore, interaction studies between the compounds indicated antagonistic effects between them. The combination of anticancer drugs may exhibit antagonistic effects, where one drug interferes with the action of another. This interference can disrupt the signaling pathways involved in caspase activation, resulting in reduced caspase enzyme activity and diminished apoptotic response. In the case of irinotecan, the addition of BCI did not reduce the drug’s activity but also did not positively affect its properties. The combination of BCI with KU60019 (ATM inhibitor) suggests potential synergy at certain concentrations of these compounds; however, the concentrations selected for cytotoxicity and apoptosis induction studies did not exhibit an intensified cytotoxic effect. Strong induction of apoptosis was only observed when the ATM inhibitor was added to the combination of irinotecan and BCI, but the results obtained in the MTT assay suggest that the combination owes this effect to the latter. Furthermore, in this system, BCI reduces the combination potential of the other two compounds. It is known that when several anticancer drugs are combined, they can interact with each other, potentially altering their mechanisms of action. This interaction can affect the ability of the drugs to induce apoptosis. It is therefore important to know the exact pathway of BCI interactions to select compounds with which it will achieve optimal effects.

## 5. Conclusions

BCI exhibits cytotoxic and proapoptotic activity in colorectal cancer cell lines (DLD1, HT-29, and Caco2) in vitro, which could potentially affect the efficiency of combination anticancer therapies. The nonspecific and DUSP6-independent cytotoxic activity can result from the inhibition of other molecular targets, e.g., CDK5, but should be investigated in vitro and in vivo.

## Figures and Tables

**Table 1:** Cell density according to cell line, time, and type of experiment.

Method	Time of Exposure	Cell Density per Well		
		DLD1	HT-29	Caco-2
MTT test	24 h	10 × 10^3^	10 × 10^3^	15 × 10^3^
	72 h	7 × 10^3^	8 × 10^3^	10 × 10^3^
Caspase 3/7 activity assay	6 h	12 × 10^3^	12 × 10^3^	15 × 10^3^
	24 h	10 × 10^3^	10 × 10^3^	12 × 10^3^
Cell cycle analysis	24 h	4 × 10^5^	5 × 10^5^	
Synergism/antagonism test	72 h	7 × 10^3^		
Flow cytometry analysis of apoptosis	24 h	4 × 10^5^		

**Table 2:** The coordinates of the grid box for all the anticancer targets used in the current study.

Drug Target	PDB Code	x-D	y-D	z-D	Spacing (Ả)	x Center	y Center	z Center
**SwissTargetPrediction**								
CASR	7dtt	78	74	126	1.000	175.726	158.479	202.05
CXCR3	6wzk	60	50	54	0.686	−22.132	−30.934	−32.037
DPP4	4pv7	50	50	50	0.369	−11.459	41.742	30.011
DRD1	7ckx	50	50	50	0.369	22.539	15.715	10.049
HTR5A	7x5h	40	40	40	0.442	109.625	133.559	116.471
ICAM1	5mza	40	40	40	0.442	5.639	1.976	−10.441
JAK1	6dbn	50	50	50	0.369	10.945	15.005	−14.57
JAK2	4c61	50	50	50	0.369	14.63	4.295	41.478
JAK3	4hvd	50	50	50	0.369	0.939	−15.305	−5.343
OPRM1	4dkl	50	50	50	0.369	−28.235	−13.287	−10.858
PRCP	3n2z	80	84	96	0.686	51.603	32.164	72.575
ROCK1	3v8s	50	50	50	0.369	−45.387	2.145	30.618
ROCK2	6ed6	50	50	50	0.369	27.148	47.046	53.015
SSTR3	7xms	108	100	96	1.000	119.725	146.465	111.596
TYK2	6dbm	50	50	50	0.369	6.261	−7.855	15.059
**SuperPred**								
APE1	6bow	66	66	66	0.731	9.292	−30.6630	−0.237
CDK1	6gu2	40	40	40	0.397	328.609	213.901	192.316
CDK5	1unl	44	44	44	0.408	59.89	28.364	27.584
CK2A1	6tls	44	44	44	0.403	77.273	7.948	21.258
CPSD	4od9	44	44	44	0.408	−3.204	12.697	−34.841
GLP-1R	5va1	100	82	108	1.000	93.575	57.237	60.469
IDO1	6e43	44	44	44	0.408	73.757	21.18	45.094
PAI-1	3cvm	88	84	98	0.631	13.584	22.736	17.343
STAT3	6nuq	44	60	44	0.403	13.619	54.024	−0.083
TOP2β	3qx3	40	40	40	0.553	32.884	95.413	50.785
TRIM24	4ybm	44	44	44	0.347	36.4360	−18.263	−32.015
**DUSP enzymes**								
DUSP2	1m3g	84	64	66	0.558	0.073	−0.088	−3.577
DUSP5	2g6z	76	78	68	0.558	27.828	100.283	28.026
DUSP6	1hzm	76	78	68	0.558	−0.49	0.023	0.053
DUSP7	4y2e	68	78	58	0.558	4.361	−1.089	3.86
DUSP8	4jmk	76	78	68	0.558	1.465	−5.469	11.323
DUSP13	5xjv	68	68	84	0.558	6.74	0.204	2.883
DUSP14	2wgp	68	76	82	0.558	−32.522	−24.129	1.42
DUSP18	2esb	50	50	50	0.353	22.401	31.495	13.982
DUSP28	5y15	68	78	68	0.558	−36.53	22.242	0.797

**Table 3:** Mean IC_50_ (±SD) values for single compounds after 72 h incubation of DLD1 cells.

Cytotoxicity IC_50_ [µM] (±SD)			
BCI	Irinotecan	Oxaliplatin	KU60019
1.21 (±0.07)	6.52 (±1.38)	4.6 (±0.68)	6.53 (±1.2)

**Table 4:** Predicted molecular properties describing Lipinski’s rule of five.

Compound	Molecular Weight	Hydrogen Bond Acceptors	Hydrogen Bond Donors	Consensus Log P Value	Druglikeness (Lipinski, Ghose, Veber, Egan Rules)
BCI	353.89 g/mol	2	1	4.25	Yes, 0 violations

**Table 5:** Predicted ADMET properties of **BCI**.

Compound/Property	Gastrointestinal (GI) Absorption (SwissADME/pkCSM)	CYP1A2 Inhibitor (SwissADME/pkCSM)	CYP2C19 Inhibitor (SwissADME/pkCSM)	CYP2C9 Inhibitor (SwissADME/pkCSM)	CYP2D6 Inhibitor (SwissADME/pkCSM)	CYP3A4 Inhibitor (SwissADME/pkCSM)	Blood Brain Barrier (BBB) Permeability (SwissADME)	P-Glycoprotein Substrate (SwissADME/pkCSM)	Ames Toxicity (pkCSM)	Cardiotoxicity (hERG Inhibition) (PreADMET)	Hepatotoxicity (pkCSM)
BCI	High (94.7% absorbed)	No/Yes	Yes/No	Yes/No	Yes/Yes	Yes/No	Yes	Yes/Yes	No	Medium risk	Yes

**Table 6:** Molecular docking results of BCI together with all the reference ligands complexed with the macromolecular targets. For full names of the protein targets, please refer to the abbreviations list.

Target	PDB ID	Binding Energy Native	Binding Energy BCI
**SwissTargetPrediction**			
CASR	7dtt	-	−6.57
CXCR3	6wzk	-	−6.43
DPP4	4pv7	−7.62	−8.4
DRD1	7ckx	−8.8	−8.83
HTR5A	7x5h	−5.66	−9.26
ICAM1	5mza	−10.18	−5.28
JAK1	6dbn	−8.49	−8.71
JAK2	4c61	−7.64	−8.65
JAK3	4hvd	−6.93	−8.86
OPRM1	4dkl	−7.8	−8.72
PRCP	3n2z	-	−7.72
ROCK1	3v8s	−8.22	−9.08
ROCK2	6ed6	−10.74	−8.85
SSTR3	7xms	-	−7.55
TYK2	6dbm	−8.94	−8.62
**SuperPred**			
APE1	6bow	-	−5.98
CDK1	6gu2	−10.2	−8.23
CDK5	1unl	−7.19	−9.17
CK2A1	6tls	−6.28	−9.26
CPSD	4od9	−9.94	−8.36
GLP-1R	5va1	-	−5.01
IDO1	6e43	−14.25	−9.15
PAI-1	3cvm	-	−6.85
STAT3	6nuq	−9.16	−6.25
TOP2β	3qx3	−9.98	−6.82
TRIM24	4ybm	−5.98	−8.66
**DUSP enzymes**			
DUSP2	1m3g		−7.64
DUSP5	2g6z		−6.28
DUSP6	1hzm		−7.47
DUSP7	4y2e		−6.68
DUSP8	4jmk		−6.3
DUSP13	5xjv		−6.75
DUSP14	2wgp		−5.89
DUSP18	2esb	−6.46	−6.03
DUSP28	5y15		−6.03

**Table 7:** Binding free energy calculation for the protein–ligand complexes.

S. No.	Complex Details	ΔG_coulomb_ ^a^	ΔG_vdw_ ^b^	ΔG_covalent_ ^c^	ΔG_solv_ ^d^	ΔG_solvlipo_ ^e^	ΔG_bind_ ^f^
1.	CDK5_BCI	−4.15	−46.85	2.26	16.56	−24.09	−57.4
2.	TRIM24_BCI	−9.18	−39.13	5.09	15.32	−28.09	−59.23
3.	CK2_BCI	0.06	−28.9	1.01	18.92	−12.23	−22.62

^a^ contribution to the MM-GBSA free energy of binding from the coulomb energy; ^b^ contribution to the MM-GBSA free energy of binding from the van der Waals energy; ^c^ contribution to the MM-GBSA free energy of binding from the covalent binding; ^d^ contribution to the MM-GBSA free energy of binding from the nonpolar contribution to the solvation energy due to the surface area; ^e^ contribution to the MM-GBSA free energy of binding lipophilic binding; ^f^ free energy of binding.

## Data Availability

The data presented in this study and in the main text of this article are available on request from the corresponding author.

## Supplementary Materials

The following supporting information can be downloaded at: https://www.mdpi.com/article/10.3390/cancers15184442/s1, Figure S1: Apoptosis detection in DLD1 cells after 24-h incubation with examined compounds by using Alexa Fluor^®^ 488 Annexin V/Dead Cell Apoptosis Kit.

## Abbreviations

5-HT5a	Serotonin 5a Receptor
ABC	ATP-Binding Cassette
ADMET	Absorption, Distribution, Metabolism, Excretion, and Toxicity
ALL	Pre-B Acute Lymphoblastic Leukemia
APE1	Apurinic/Apyrimidinic Endonuclease 1
ATM	ATM Serine/Threonine Kinase
BBB	Blood–Brain Barrier
BCI	(E)-2-benzylidene-3-(cyclohexylamino)-2,3-dihydro-1H-inden-1-one
CASR	Calcium Sensing Receptor
CDK1	Cyclin-Dependent Kinase 1
CDK5	Cyclin-Dependent Kinase 5
CHK1/2	Checkpoint Kinases 1/2
CK2A1	Casein Kinase II Subunit Alpha
CNS	Central Nervous System
CPSD	Cathepsin D
CRC	Colorectal Cancer
CXCR3	C-X-C Chemokine Receptor Type 3
CYP	Cytochrome
DDR	DNA Damage Response
DPP4	Dipeptidyl Peptidase IV
DRD1	Dopamine D1 Receptor
DUSP	Dual Specificity Phosphatase
DUSP6	Dual Specificity Phosphatase 6
EGFR	EGFR: Epidermal Growth Factor Receptor
ERK	Extracellular Signal-Regulated Kinase
GI	Gastrointestinal
GLP-1R	Glucagon-like Peptide-1 Receptor
hERG	Human Ether-a-go-go-Related Gene
ICAM-1	Intercellular Adhesion Molecule
IDO1	Indoleamine 2,3-Dioxygenase-1
IND	Investigational New Drug
IR	Ionizing Radiation
JAK1/2/3	Tyrosine-Protein Kinase JAK1/2/3
LQTS	Long QT Syndrome
MAPKs	Mitogen-Activated Protein Kinases
MKP	Mitogen-Activated Protein Kinases Phosphatases
MPNST	Malignant Peripheral Nerve Sheath Tumor
NC	Negative Control
OPRM1	Mu Opioid Receptor
PAI-1	Plasminogen Activator Inhibitor Type-1
PRCP	Lysosomal Pro-X Carboxypeptidase
RMSD	Root-Mean-Square Deviation
RMSF	Root-Mean-Square Fluctuation
ROCK1/2	Rho-Associated Protein Kinase-1/2
ROS	Reactive Oxygen Species
SOD2	Superoxide Dismutase 2
SSE	Secondary Structure Analysis
SSTR3	Somatostatin Receptor 3
STAT3	Signal Transducer and Activator of Transcription-3
TNF-α	Tumor Necrosis Factor α
TOP2β	Topoisomerase-II Beta
TP53	Cellular Tumor Antigen p53
TRIM24	Tripartite Motif-Containing 24
TYK2	Tyrosine-Protein Kinase TYK2
