# Supplementary material for: In Vitro and In Silico Investigation of BCI Anticancer Properties and Its Potential for Chemotherapy-Combined Treatments
Source: Cancers (Basel). 2023 Sep 6;15(18):4442. doi: 10.3390/cancers15184442 (PMC10526149; doi:10.3390/cancers15184442)
Supplement: Supplementary file 1 [file cancers-15-04442-s001.zip › cancers-2540884-supplementary.pdf]

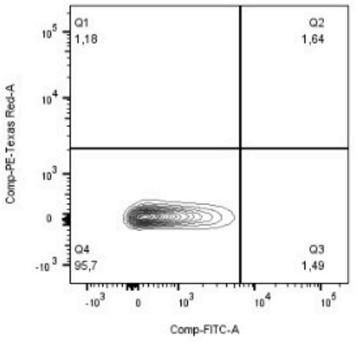

Control 1

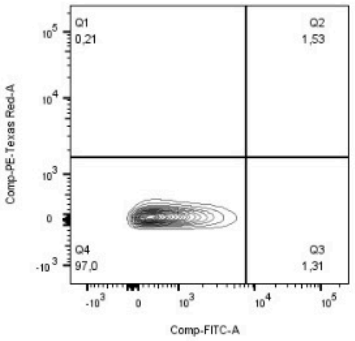

Control 2

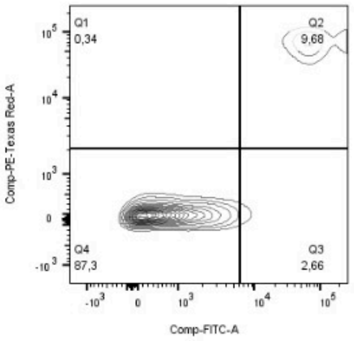

Control 3

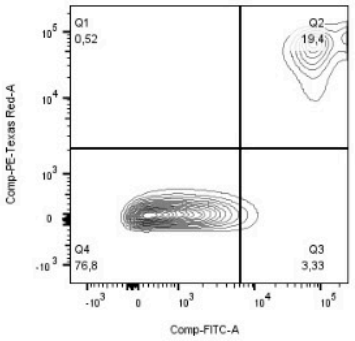

BCI 1

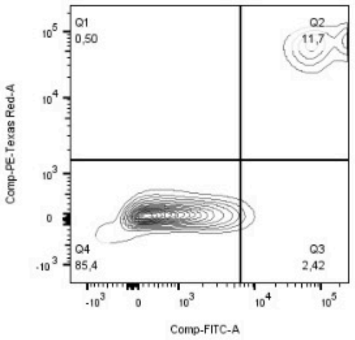

BCI 2

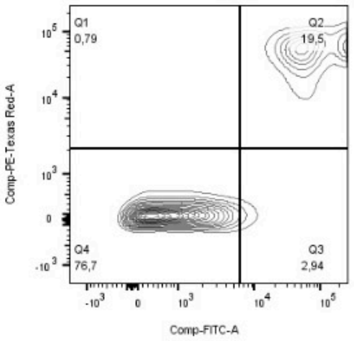

BCI 3

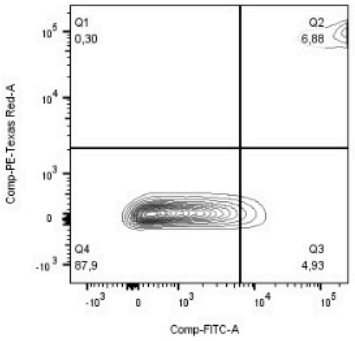

Irtb 1

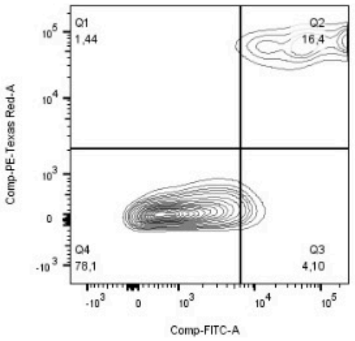

Irtb 2

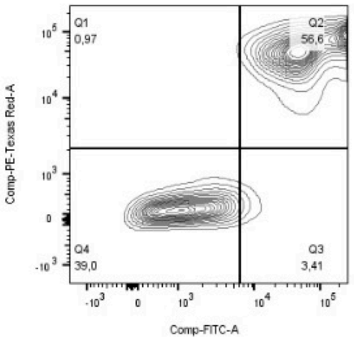

Irtb 3

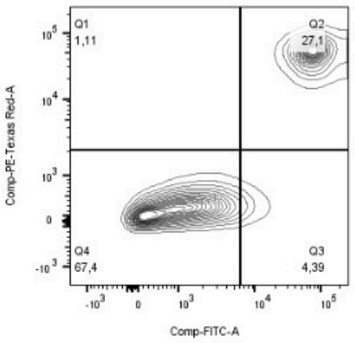

Oxpt 1

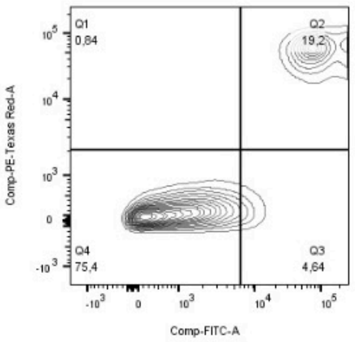

Oxpt 2

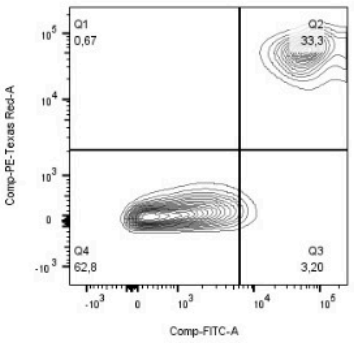

Oxpt 3

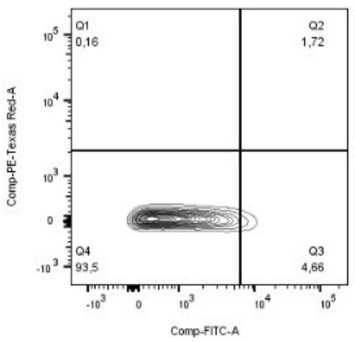

KU 1

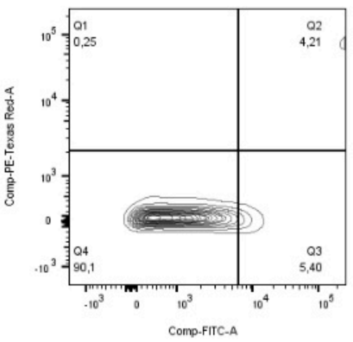

KU 2

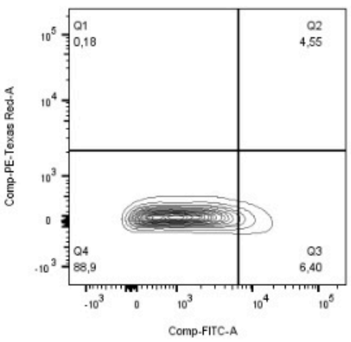

KU 3

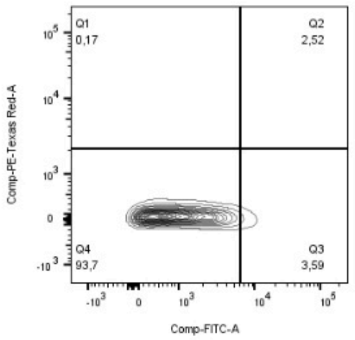

KU+BCI 1

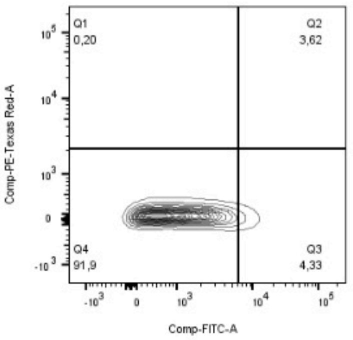

KU+BCI 2

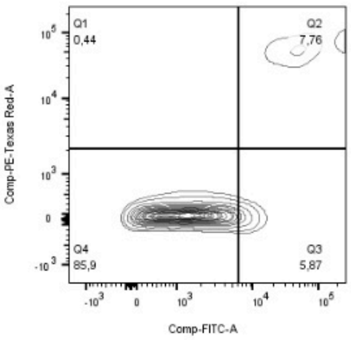

KU+BCI 3

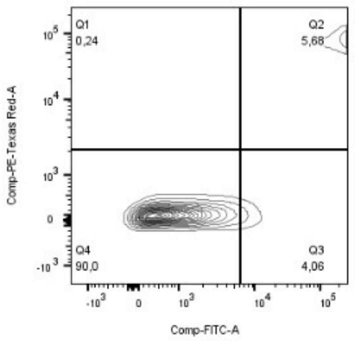

Oxpt+BCI 1

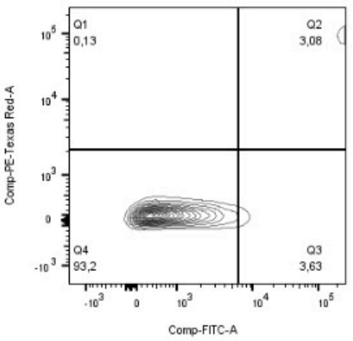

Oxpt+BCI 2

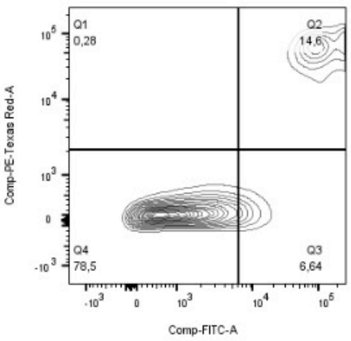

Oxpt+BCI 3

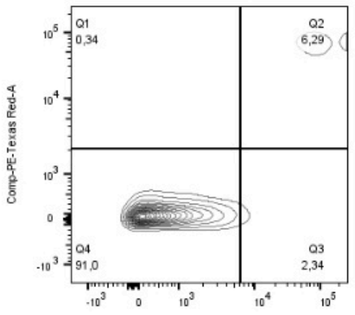

Irtb+BCI 1

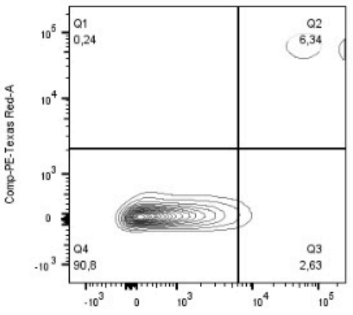

Irtb+BCI 2

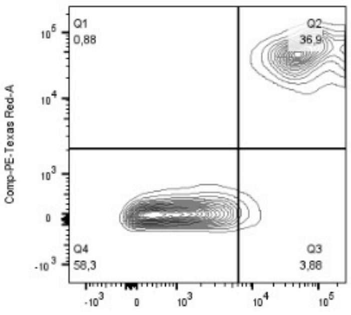

Irtb+BCI 3

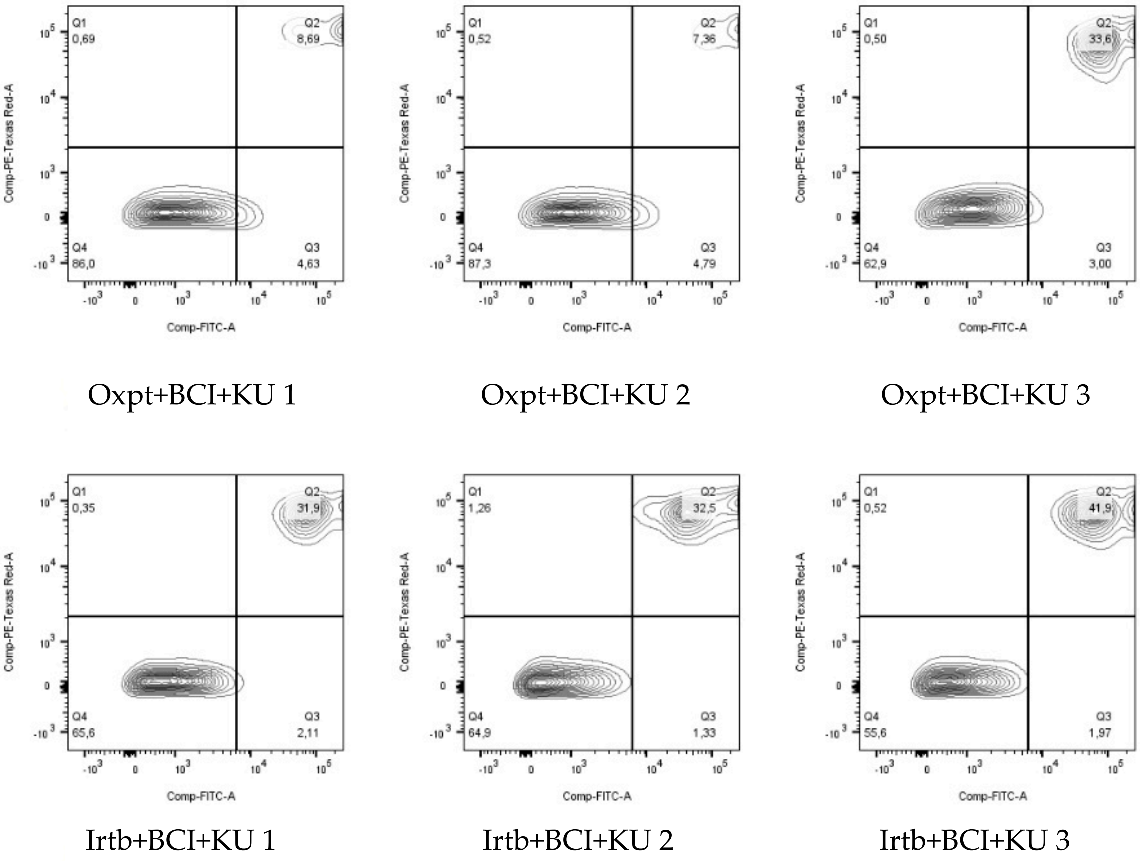

Figure S1. Apoptosis detection in DLD1 cells after 24-h incubation with examined compounds by using Alexa Fluor® 488 Annexin V/Dead Cell Apoptosis Kit. The distribution of DLD1 cells classified as necrotic (Q1), early apoptotic (Q4), late apoptotic (Q2), and viable cells (Q3)
